# Evaluating Magnetic Seed Localization in Targeted Axillary Dissection for Node-Positive Early Breast Cancer Patients Receiving Neoadjuvant Systemic Therapy: A Comprehensive Review and Pooled Analysis

**DOI:** 10.3390/jcm13102908

**Published:** 2024-05-14

**Authors:** Munaser Alamoodi, Umar Wazir, Rita A. Sakr, Janhavi Venkataraman, Kinan Mokbel, Kefah Mokbel

**Affiliations:** 1 London Breast Institute, The Princess Grace Hospital, 42-52 Nottingham Place, London W1U 5NY, UK; malamoodi@kau.edu.sa (M.A.); umar.wazir@rcsed.ac.uk (U.W.); janhavi.venkataraman@hcahealthcare.co.uk (J.V.); k.mokbel@ex.ac.uk (K.M.); 2 Department of Surgery, King Abdulaziz University, Jeddah 21589, Saudi Arabia; 3 College of Medicine, University of Sharjah, Sharjah 27272, United Arab Emirates; dr.ritasakr@gmail.com; 4 Department of Oncoplastic Surgery, King’s College Hospital London, Dubai P.O. Box 340901, United Arab Emirates; 5 Health and Care Profession Department, College of Medicine and Health, University of Exeter Medical School, Exeter B3183, UK

**Keywords:** neoadjuvant systemic therapy, breast cancer, targeted axillary lymph node, magnetic seed, pathological complete response

## Abstract

**Background/Objectives:** De-escalation of axillary surgery is made possible by advancements in both neoadjuvant systemic therapy (NST) and in localisation technology for breast lesions. Magseed^®^, developed in 2013 by Dr. Michael Douk of Cambridge, United Kingdom, is a wire-free localisation technology that facilitates the localisation and retrieval of lymph nodes for staging. Targeted axillary dissection (TAD), which entails marked lymph node biopsy (MLNB) and sentinel lymph node biopsy (SLNB), has emerged as the preferred method to assess residual disease in post-NST node-positive patients. This systematic review and pooled analysis evaluate the performance of Magseed^®^ in TAD. **Methods:** The search was carried out in PubMed and Google Scholar. An assessment of localisation, retrieval rates, concordance between MLNB and SLNB, and pathological complete response (pCR) in clinically node-positive patients post NST was undertaken. **Results**: Nine studies spanning 494 patients and 497 procedures were identified, with a 100% successful deployment rate, a 94.2% (468/497) [95% confidence interval (CI), 93.7–94.7] localisation rate, a 98.8% (491/497) retrieval rate, and a 68.8% (247/359) [95% CI 65.6–72.0] concordance rate. pCR was observed in 47.9% (220/459) ) [95% CI 43.3–52.6] of cases. Subgroup analysis of studies reporting the pathological status of MLNB and SLNB separately revealed an FNR of 4.2% for MLNB and 17.6% for SLNB (*p* = 0.0013). Mean duration of implantation was 37 days (range: 0–188). **Conclusions:** These findings highlight magnetic seed localisation’s efficacy in TAD for NST-treated node-positive patients, aiding in accurate axillary pCR identification and safe de-escalation of axillary surgery in excellent responders.

## 1. Introduction

Due to the significant morbidity associated with it, complete axillary lymph node dissection (ALND), in which a group of lymph nodes is removed from the axilla of a patient with breast cancer to assess the extent of cancer spread and to remove potentially cancerous lymph nodes for further examination, has largely been supplanted by the less-invasive sentinel lymph node biopsy (SLNB) as the gold standard for regional axillary staging in clinically node-negative breast cancer patients undergoing upfront surgery or staging the axilla in patients with cN0 breast cancer after neoadjuvant systemic therapy (NST) where chemotherapy, hormone therapy, or targeted therapy is given before surgery or other primary treatments [1].

Recent trials, including After Mapping of the Axilla: Radiotherapy Or Surgery trial (AMAROS, 2023) [2] and the American College of Surgeons Oncology Group Z0011 trial (ACOSOG Z0011, 2017) [3], have shown that skipping ALND in patients with a positive SLNB does not impact overall survival (OS). These findings have sparked interest in broadening the scope of axillary surgery de-escalation to include patients with cN1 breast cancer who respond favourably to NST. However, in biopsy-proven node-positive patients undergoing NST, studies on SLNB have shown inconsistent rates of false negatives and identification [4]. Our recent meta-analysis (2016), encompassing over 3000 patients with breast cancer involving the axillary lymph nodes, revealed a false negative rate (FNR) of 13% after NST exceeding the safe threshold of 10% [4].

The studies included in our meta-analysis were retrospective in nature, displayed significant heterogeneity, and lacked standardisation of protocols. The relatively high FNRs associated with SLNB post NST has been attributed to anatomical changes leading to altered lymphatic drainage, NST-induced fibrosis, fat necrosis, granulation tissue formation, or characteristics of the tumour itself. Hence, the subsequent logical progression was to investigate the potential integration of postoperative pathological evaluation of the biopsy-proven lymph node that had been tagged before NST. We have shown that incorporating the marked lymph node biopsy, whereby a biopsy-proven clipped node is marked with a localisation technique such as Magseed (MLNB) alongside SLNB in this context, is linked to an acceptably low FNR of 5.18% (95% CI: 3.41–7.54) [5].

Until recently, axillary lymph node dissection (ALND) was the standard approach for managing the axilla in patients with clinically positive lymph nodes (cN+), irrespective of their response to neoadjuvant systemic therapy (NST) [1]. However, the landmark Z0011 trial [3] demonstrated that early-stage breast cancer patients with low nodal burden on sentinel lymph node biopsy (SLNB) did not have inferior outcomes compared to those who underwent ALND. These findings do not support routine use of ALND in this population based on 10-year outcomes. Despite this, concerns have arisen regarding the oncological safety of SLNB due to its high false-negative rate (FNR) in patients initially cN+ rendered ypN0 post-NST. The FNR ranged between 11.9 and 14.2 higher than the accepted rate of 10% which is considered oncologically safe [2,3,4,5].

Pathological complete response (pCR) is defined as the absence of any residual invasive cancer in the breast and/or lymph nodes following NST. Achieving pCR in the breast often correlates with axillary pCR, prompting consideration for de-escalating ALND in responders to minimise morbidity and improve quality of life [6,7]. Particularly, locally advanced and biologically aggressive breast cancers such as triple-negative and HER2-positive subtypes exhibit high pCR rates post NST [8].

To address the need for precise localisation of tumour-involved lymph nodes while minimising surgical invasiveness, novel wire-free technologies such as Magseed^®^ have been developed. Magseed^®^ is a nonradioactive ferromagnetic marker containing iron particles. A handheld magnetometer called Sentimag was developed specifically for this purpose. The Sentimag probe generates an alternating magnetic field that briefly magnetises the iron in Magseed^®^. The Sentimag probe detects the small magnetic signature produced by Magseed^®^. Measuring 5 × 1 mm in length, Magseed^®^ can be deployed under mammography, ultrasound, or computed tomographic guidance using a preloaded sterile 18-gauge needle and can be detected within 4 cm depth from the skin’s surface [9,10]. It is used to mark breast lesions and/or lymph nodes preoperatively. Intraoperatively, the probe detects the seed placed in the lesion, accurately directing the surgeon to the lesion and facilitating the retrieval of the lesion for pathological assessment. However, challenges arise in localising biopsy-proven lymph nodes post NST, as they may normalise and become difficult to identify on imaging [5,11,12]. A node containing the seed is termed a marked lymph node (MLN). In addition to magnetic seeds, other wire-free methods for marking pathological lymph nodes, such as iodine-125 (^125^I) seeds, electromagnetic reflectors, or radio-frequency tags can be combined with SLNB in an operative strategy termed targeted axillary dissection (TAD) in the hope to reduce the FNR associated with SLNB alone after NST for node-positive breast cancer [5,13].

While ^125^I radioactive seed localisation (RSL) is commonly used for axillary node localisation, its strict handling regulations and signal decay over time pose limitations [13]. In contrast, Magseed^®^ offers advantages as a non-radioactive alternative, with no signal decay and being certified as safe for prolonged implantation by the Federal Drug Administration (FDA), allowing for pre-NST placement [13]. This systematic review assesses Magseed’s performance in TAD and its efficacy in patients undergoing NST for node-positive early breast cancer.

## 2. Materials and Methods

### 2.1. Literature Search

This study received approval from the multidisciplinary breast cancer board of the London Breast Institute. A comprehensive literature search was conducted using PubMed and Google Scholar databases up to February 2024. The search utilised the following keywords:

- [magnetic seed] OR [Magseed];
- [targeted axillary dissection] OR [TAD];
- [breast cancer];
- [neoadjuvant].

Additionally, bibliographies of relevant studies were examined for potential inclusion. A significance threshold of *p* < 0.05 was applied for statistical analyses.

### 2.2. Inclusion and Exclusion Criteria

Studies identified in the literature search were evaluated based on the following inclusion and exclusion criteria.

#### 2.2.1. Inclusion Criteria

Studies were included if they met the following criteria:

- Retrospective or prospective cohort design;
- Investigation of the role of magnetic seeds in TAD in patients undergoing neoadjuvant systemic therapy (NST);
- Availability of data endpoints, including successful localisation and retrieval rate, SLNB-MLNB concordance rate, pathological complete response (pCR), and migration rate.

#### 2.2.2. Exclusion Criteria

Studies meeting the following criteria were excluded:

- Manuscripts not available in English;
- Studies involving non-human subjects;
- Non-peer-reviewed studies;
- Studies with 10 or fewer eligible cases;
- Case reports.

#### 2.2.3. Statistical Analysis

The statistical analysis software ‘STATA’ (STATA Corporation, College Station, TX, USA) was employed to assess heterogeneity and produce a forest plot for successful localisation endpoints. GraphPad software was used to determine 95% confidence intervals. MedCalc software was used to conduct a chi-square test.

Statistical results were considered significant if the *p*-value was less than 0.05.

## 3. Results

### 3.1. Literature Search Results

**Figure 1:**
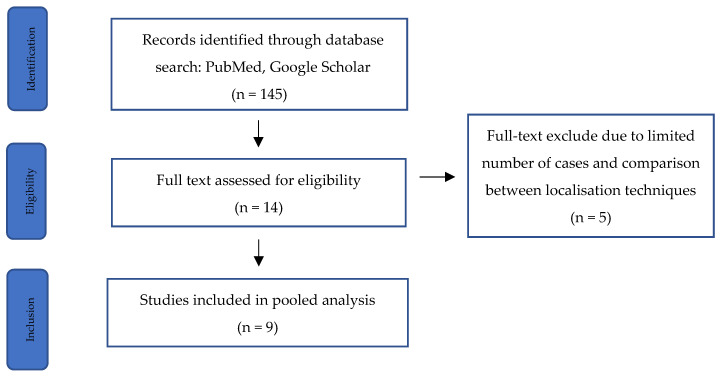
PRISMA flow diagram illustrating the inclusion and exclusion of studies.

The search yielded 145 articles, of which 9 met the inclusion criteria, encompassing 494 patients (Table 1; Figure 1) [14,15,16,17,18,19,20,21,22].

### 3.2. Pooled Analysis

Nine studies involving 494 patients (497 procedures) met the inclusion criteria. The pooled average age was 53 years (range: 17–92). The pooled analysis revealed the following:

Successful localisation rate: 94.2% (468/497) [95% confidence interval (CI), 93.7–94.7];

Retrieval rate: 98.8% (491/497) [95% CI: 97.3–99.6];

Concordance rate between SLNB and MLNB: 68.8% (247/359) [95% CI 65.6–72.0]. Subgroup analysis of studies reporting the pathological status of MLNB and SLNB separately revealed an FNR of 4.2% for MLNB and 17.6% for SLNB (*p* = 0.0013; chi-square statistic).

pCR was observed in 47.9% of cases (220/459) [95% CI 43.3–52.6], with no reported migration or procedure-specific complications.

In one study, the Magseed was not retrieved, possibly lost during suction in the operating theatre. No significant artefact was observed on postoperative MRI [22].

The successful deployment rate was 100%, but one patient required repeat deployment due to seed misplacement during ultrasound-guided localisation [22].

In another study, localisation was compromised in two patients due to the inability to visualise the clip by ultrasound, and a technical difficulty led to malposition in one patient [21].

The pooled average number of lymph nodes retrieved in the TAD procedure was 1.8 (range: 1–11).

The pooled average duration from magnetic seed deployment to surgery was 37 days (range: 0–188 days).

**Figure 2:**
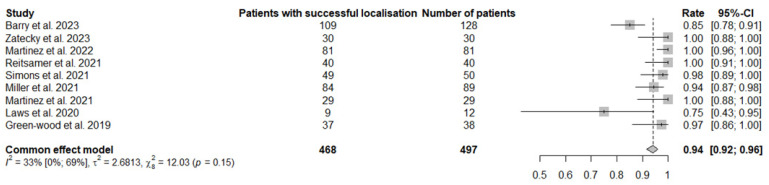
Forest plot of successful localisation rate in the pooled analysis [14,15,16,17,18,19,20,21,22].

There was no significant heterogeneity among the studies analysed (I^2^ = 33%, *p* = 0.15). Figure 2 shows a forest plot of the pooled analysis for the successful localisation rate.

## 4. Discussion

### 4.1. Performance of Magseed^®^ in Targeted Axillary Dissection (TAD)

**Figure 3:**
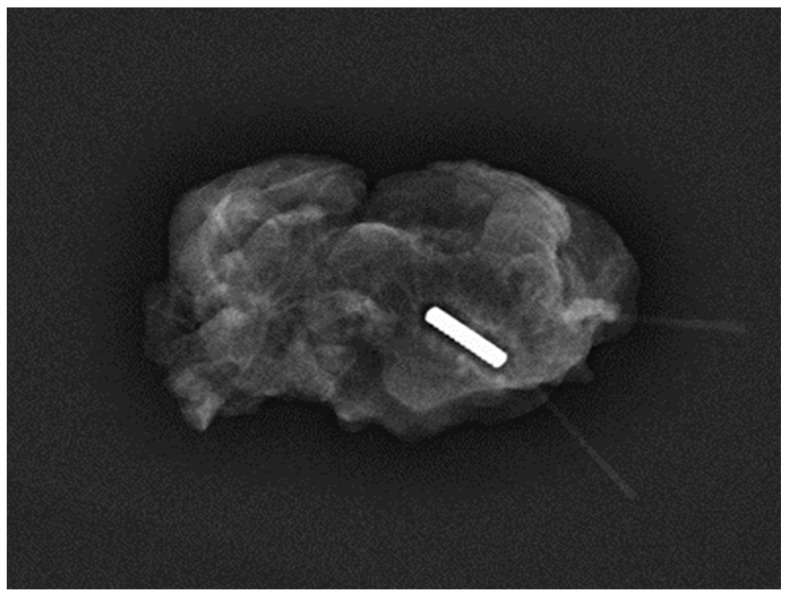
Specimen radiograph of a magnetic seed containing a lymph node harvested during TAD in a 65-year-old patient who received NST after the deployment of the seed (Magseed^®^).

Magseed^®^ technology, introduced into clinical practice in 2016, has undergone improvements to enhance user-friendliness and MRI compatibility, expanding its utility [22]. Initially utilised primarily for non-palpable breast lesions [23], its acceptance for axillary applications has gradually increased. However, there remains limited literature on its efficacy in aiding TAD. Our analysis, encompassing 494 Magseed^®^ TAD procedures, provides evidence of its performance since the publication of the first study by Greenwood et al. in 2019. The 100% successful deployment rate is facilitated by the small size of the seed (5 × 1 mm) and narrow bore of the introducer needle (18 gauge) [23] (Figure 3).

Our analysis confirmed high successful localisation (94.2%) and retrieval rates (98.8%), indicating Magseed^®^’s excellent efficacy in aiding TAD. Notably, the successful localisation rate of 94.2% compares favourably with that reported in our previously pooled analysis (90%) encompassing various technologies and involving 1470 marked lymph nodes [23].

Our analysis showed a limited concordance rate of 68.8% between the SLNB and MLNB, underscoring the need to include both components in TAD to accurately stage the axilla post NST. The FNR of the SLNB (17.6%) alone is aligned with previous studies and is above the acceptable threshold of 10% [4].

The deployment of Magseed^®^ pre NST demonstrated significantly higher success rates compared to post-NST deployment, attributed to technical challenges in localising treated lymph nodes post NST, as they can often shrink significantly and consequently are difficult to visualise [14]. The single-stage approach of deploying the magnetic seed at the time of biopsy is also more cost-effective. Magseed^®^’s approval for long-term use in soft tissue enables pre-NST deployment, streamlining the localisation process and avoiding additional patient visits. Importantly, successful localisation is crucial for accurate pathological assessment and subsequent treatment decisions. The low FNR of TAD compared with ALND underscores its potential oncological safety and its role in identifying patients responding favourably to NST.

### 4.2. Comparison of Wireless Technologies for Localisation

**Figure 4:**
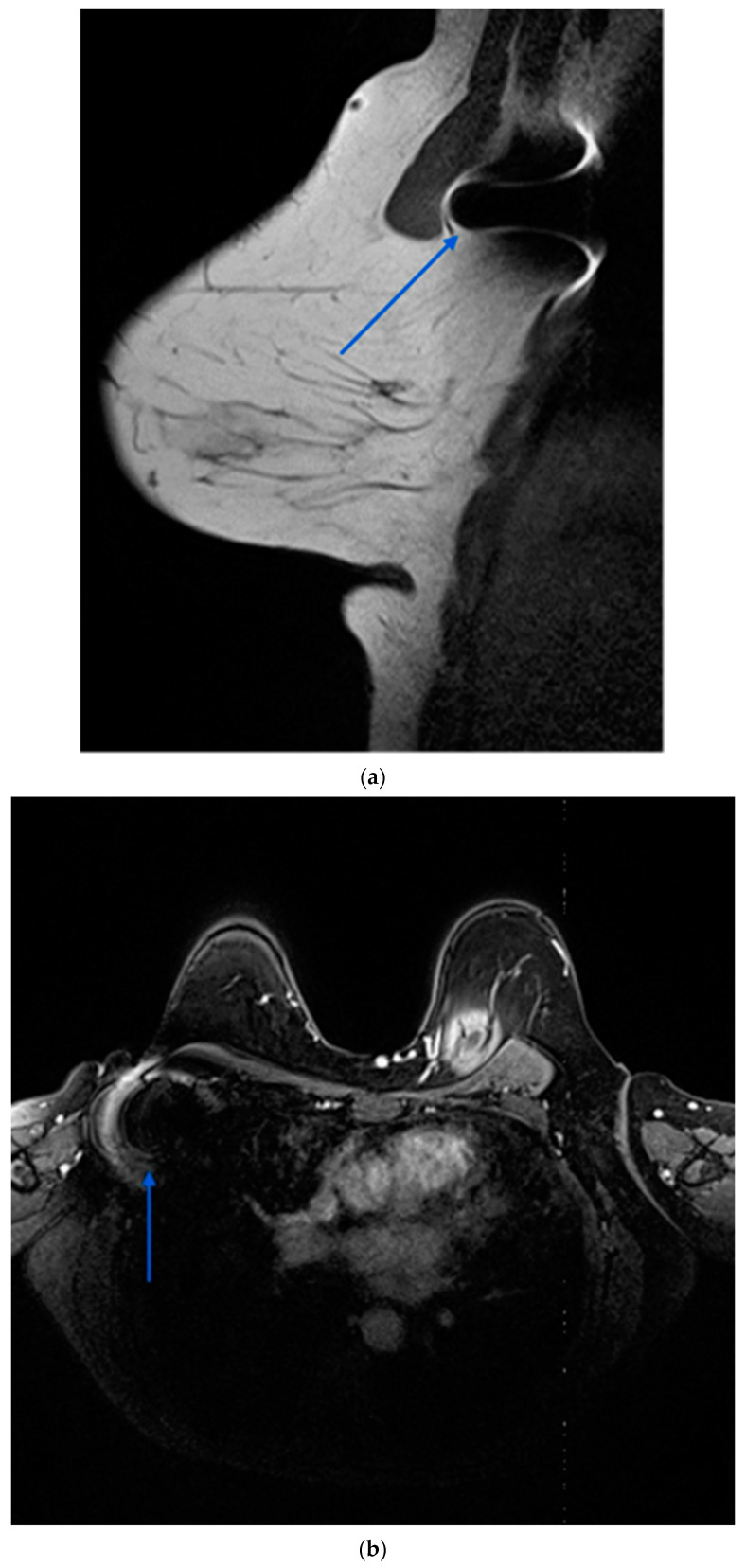
Contrast enhanced MRI after completion of NST demonstrating ferromagnetic artefact (blue arrows) generated by a magnetic seed previously placed in a right pathological axillary lymph node. (**a**): sagittal view; (**b**): axial view.

Several localisation technologies, including RSL, magnetic seeds (Magseed^®^), radio-frequency identification (RFID) tags, and SAVI SCOUT, offer advantages and limitations. Magseed^®^, a non-radioactive wireless marker, demonstrates high localisation rates and can be deployed at the time of biopsy, facilitating streamlined surgical procedures. However, it entails higher costs and may interfere with MRI interpretation due to the generation of significant void signals up to 4 cm (Figure 4) [5,24].

RSL is cost-effective when compared to other wireless marking techniques but requires radioactive handling and strict regulatory compliance. Furthermore, the marker can be safely implanted for up to 7 days only, limiting the flexibility of surgical scheduling [25,26].

RFID tags offer wireless deployment but may suffer from migration and create interference artefacts in MRI scans [27].

SAVI SCOUT, another non-radioactive wireless option, provides accurate localisation but is larger than Magseed^®^ and lacks flexibility in marker adjustment post deployment [28].

A prominent limitation of the Magseed® system would be the requirement to remove all metal instruments from the surgical field during the use of the Sentimag probe. Furthermore, the ferromagnetic seeds interfere with MRI creating significant artefacts that can compromise the assessment of response to NST in both the axilla and the lateral aspect of the breast [29,30]. Additional drawbacks of this technology involve the necessity for frequent recalibration of the Sentimag due to interference from paramagnetic equipment, as well as its limited depth for accurate detection, which extends only up to 30 mm.

Table 2 summaries the features of the main wire-free technologies.

### 4.3. Imaging Modalities

While imaging modalities play a crucial role in BC diagnosis and staging, their efficacy in assessing residual axillary lymph node disease post NST is limited. Current non-invasive imaging modalities exhibit suboptimal accuracy in detecting residual disease, highlighting the necessity for axillary procedures post NST. A recent meta-analysis studying the sensitivity, specificity, positive predictive value (PPV), and negative predictive value (NPV) of current modalities, including ultrasound, MRI, and 18F-FDG PET-CT, found values considerably below those for surgical biopsy procedures [34].

Some new modalities show promise. De Mooij et al. reported on a pilot study exploring the potential role of hybrid PET-MRI in predicting pCR preoperatively and observed a PPV of 100% and NPV of 67%. The NPV rose to 90% after excluding clinically inert micro-metastases [35].

### 4.4. The Role of Artificial Intelligence (AI)

The integration of AI into imaging modalities is currently a very active area of development in the treatment of breast cancer. Computer-assisted detection (CAD) has been approved for use in the screening and diagnosis of breast cancer since 1998 [36,37], and has achieved an uptake of 92% in US centres [38]. However, CAD has been associated with added costs to imaging and high false-positive rates in reading mammograms, which has caused controversy regarding its utility [39]. More recently, deep learning and convolutional neural networks (CNNs) have reignited the interest in the development of AI tools to assist in imaging [40]. As of 2022, nine AI products have received FDA approval for use in breast screening [36].

Imaging-related AI tools either assist with workflow or assist with detection. Li et al. reported using an AI-assisted diagnosis system with 18F-FDG PET-CT in 407 patients with breast cancer. They found that the AI tool increased clinician sensitivity by 2–4% [41]. Tahmasebi et al. studied the performance of three AI models powered by AutoML (Alphabet Inc., Mountain View, CA, USA) on a set of 64 ultrasound scans of patient’s axillae and compared their performance with that of three radiologists. They found that the performance of human radiologists and the AI models was comparable, with higher sensitivity for the human assessors, and higher specificity for the AI models [42]. Ge et al. tested an AI-enabled workflow to generate personalised ultrasound reports for breast patients and trained it on 4809 patients. They found it increased physicians’ work efficiency by 90% [43]. Zhang et al. described an AI assistant that increased the efficiency of annotating breast MRIs by a factor of 20 [44].

The development of AI tools is becoming an area of rapid development in the current era which will need to be revisited often.

### 4.5. Oncological Safety of Targeted Axillary Dissection (TAD)

One of the predominant trends in modern oncological practice has been the incremental de-escalation of surgical treatment of breast cancer. Modified radical mastectomy has given way to wide local excision as the preferred surgical approach towards the primary tumour in early breast cancer [45]. Similarly, SLNB represents a significant de-escalation in the approach to axillary staging and treatment of axillary disease when compared to the gold-standard practice of the past [1,2]. This has all been possible because of evidence of the safety of de-escalation furnished by multiple seminal clinical trials [46].

This trend towards de-escalation has continued in more niche clinical scenarios, such the treatment of axillary disease in node-positive patients after NST. In terms of survival, currently available evidence contends that there is no difference between SLNB and ALND after the completion of NST [47,48]. However, there are concerns regarding the reliability of SLNB as a staging modality in node-positive patients who undergo NST. As per a systematic review by El Haj et al., the FNR of such an SLNB could be as high as 13% [4]. Indeed, it was such concerns which lead to the development of TAD, in which SLNB is combined with MLNB. In their initial description, Caudle et al. reported an FNR of 2% for TAD [13].

Since the original description of the procedure in 2016 [13], evidence of the oncological safety of TAD has been gradually accumulating. Kuemmel et al. reported a prospective study including 199 patients comparing survival in patients undergoing TAD alone and TAD combined with ALND. After 43 months of follow-up, similar recurrence and survival was seen in both groups if at least three nodes were harvested in TAD [49]. Similarly, Wu et al., while reporting on a prospective registry study comparing TAD and ALND, found similar outcomes in both groups in terms of survival and disease recurrence [50].

A further piece of evidence in favour of de-escalation of axillary surgery is the recently presented 5-year data of the NSABP B-51 trial, which included patients who received NST for cN1 disease and were down-staged to ypN0 and compared outcomes in patients who received regional nodal irradiation (RNI) with those who did not. The data presented at the 2023 San Antonio Breast Cancer Symposium by the trial authors suggest that RNI could be safely omitted in such cases [51]. When taken with the conclusions of the AMOROS trial, which found similar survival outcomes in patients undergoing ALND and RNI [2], a robust case could be made for de-escalation of axillary surgery in patients who respond well to NST. In patients with N2-3 disease, a recent prospective registry study involving 218 patients undergoing NST and TAD according to the MARI protocol showed that omission of ALND in selected patients who attained pCR and received radiation treatment was associated with excellent oncologic outcomes after a median follow-up of 44 months. The next crucial step is to carry out trials that would involve replicating the B-51 trial to evaluate whether RNI can also be safely omitted in addition to ALND in pCR cases within the context of cN2-3 disease [52]. It should be noted that although both studies were presented at international conferences and their abstracts have been published, the peer-reviewed full articles have not been published yet.

The adoption of TAD has been increasing of late. A survey conducted by the Northeastern German Society of Gynaecological Oncology (NOGGO) to find out the attitudes of surgeons/radiologists towards TAD revealed widespread utilisation of TAD in patients converting to ycN0 post NST. However, the survey also highlighted a heterogeneous approach to axillary management, suggesting the need for comparative evaluations of different marking techniques [53]. A total of 116 physicians completed the survey. TAD was used at the departments of 82% of respondents and was offered to all cN+ converting to ycN0 by 57% and only to selected patients. The most common marking was clip or coil. The survey highlights the need for further studies such as this one to introduce more physicians to these novel marking techniques. Furthermore, TAD is cited as a superior alternative to SLNB in post-NST patients who presented with node-positive disease in the 2022 NCCN Clinical Practice Guidelines for breast cancer [54]

### 4.6. Limitations

This study collates data from the current literature regarding the use of Magseed^®^ in TAD and is a useful starting point for discussions regarding the use of this novel technology in this clinical role. However, the limitations of this study have to be acknowledged. At this early stage, the available studies are limited, and the data used in this study include retrospective as well as prospective studies. Some of the studies used have been carried out in a single institution and can only reflect on the learning experience of that institution. In most of the studies used, there was no survey on patient satisfaction or economic analysis. Hopefully, the availability of more prospective studies in the future would enable more complete systemic reviews from which conclusion could be drawn with more confidence.

Although the data presented relate to Magseed^®^, it is worth noting that there are other systems that utilise magnetic seeds for localisation, such as the magnetic marker localisation (MaMaLoc, Sirius Medical Systems B.V., Eindhoven., The Netherlands) system. However, there are currently no data available regarding the use of MaMaLoc in TAD [55].

## 5. Conclusions

Magnetic seed localisation demonstrates efficiency in TAD for NST-treated patients with positive nodes, facilitating accurate axillary pCR identification and safe omission of ALND in terms of survival and decreased associated morbidity in strong responders. Furthermore, accurate cancer staging after NST can facilitate risk-adapted treatment optimisation. The substantial MRI artefacts generated by magnetic seeds represent a significant limitation in the context of an optimal TAD approach.

## Figures and Tables

**Table 1:** NST: neoadjuvant systemic therapy, pCR: pathological complete response, SLNB: sentinel lymph node biopsy, MLNB: marked lymph node biopsy.

Study	Citation	Number of Patients	Mean Age (Years)	pCR	Retrieval Rate	Localisation Rate	Migration Rate	Mean Implantation Duration (Days)	Median Number of Nodes Harvested	SLNB-MLNB Concordance Rate	FNR of MLNB	FNR of SLNB
Barry et al. (2023) (Retrospective)	[14]	128 (9 × 2 magseeds) (74 post and 54 pre-NST)	59.1	75/128 (59%)	128/128 (100%)	(Post-NST 56/74 = 75.7%) (pre-NST 53/54 = 98.2%)	-	20 (89–188)	2	75/128 (59%)	5/53 (9.4%)	12/53 (22.6%)
Zatecky et al. (2023) (Prospective)	[15]	30	49.4 (26–80)	12/30 (40%)	30/30 100%	30/30 (100%)	0	138.5	3.5 (1–10)	25/30 (83.3)	0/18	-
Martinez et al. (2022) (Prospective)	[16]	81 (37 post- and 44 pre- NST)	47 (29–78)	37/81 (45.6%)	81/81 100%	81/81 (100%)	0	-	1 (1–8)	66/81 (81.5%)	0/45	5/45 (11.1%)
Reitsamer et al. (2021) (Prospective)	[17]	40	52 (29–81)	27/40 (67.5%)	40/40 (100%)	40/40 (100)	0	-	2.3 (1–9)	26/40 (65%)	0/13	2/13 (15.4%)
Simons et al. (2021) (Retrospective)	[18]	50		22/50 (44.0%)	50/50 (100%)	49/50 (98%)	-	0–30	1.3 (1–6)	40/50 (80%)	-	-
Miller et al. (2021) (Retrospective)	[19]	89	58 (17–92)	27/89 (30%)	84/89 (94.1%)	84/89 (94.1%)	-	-	-	-	-	-
Mariscal Martinez et al. (2021) (Prospective)	[20]	29 (1 patient bilateral)	55 (30–78)	14/29 (48.3%)	29/29 100%	29/29 (100%)	-	10 (1–26)	1.2 (0–2)	15/30 (50%)	1/14	3/14
Laws et al. (2020) (Retrospective)	[21]	12	51 (30–73)	6/12 (50%)	12/12 (100%)	9/12 (75%)		(0–22)	3 (1–11)			
Greenwood et al. (2019) (Retrospective)	[22]	35 (38 localisation)	56 (32–78)		37/38 (97%)	37/38 (97%)		5 (0–31)				
Total		494 (497 procedures)	53 (17–92)	220/459 (47.9%)	491/497 (98.8%)	468/497 (94.2%)	0	37(0–188)	1.8 (1–11)	247/359 (68.8%)	6/143 (4.2%)	22/125 (17.6%)

**Table 2:** Comparison of wireless technologies.

Localisation System	Salient Features	Advantages	Disadvantages
Radioactive seed localisation (RSL)	Seed placed in the lesion and accurately detected with handheld gamma probe [25].	Cost-effective and can improve oncological outcomes of image-guided surgery [31]. TAD with ^125^I achieved 99.3% identification rate [26].	Placed 5–7 days before surgery [25]. Radioactive. Requires licensing and strict regulatory requirements
Magnetic seed (Magseed; Endomagnetics Inc., Cambridge, UK) [24].	Inducible ferromagnetic seed made of surgical-grade stainless steel. Reliable detection at 4 cm depth, can be up to 12 cm according to manufacturer. Sentimag probe induces and detects the magnetic field of a Magseed with audio signal [10].	Non-radioactive, wireless, and can be deployed at the time of biopsy. Localisation 99.86% [23]. TAD with Magseed localisation achieved 96.0% in the AXANA trial [29]. Audio reading only.	High cost. May interfere with MRI. Inability to adjust the position of the marker once deployed. Limitations in deep (>6 cm) non-palpable lesions [32]. Necessitates the removal of all metal equipment before localisation [30].
Radiofrequency identification (RFID) tags (LOCalizer; Hologic, Santa Carla, CA, USA) [27].	Ferrite rod covered with copper and wrapped with a microprocessor and glass casing detected by a radiofrequency reader [30].	Non-radioactive wireless. Can be deployed at the time of biopsy. Audiovisual reading. 91.0% retrieval rate [21].	High cost. Utilises a wider introducer than Magseed. Can interfere with MRI. Susceptibility to migration [33].
SAVI SCOUT (Cianna Medical Inc., Aliso Viejo, CA, USA) [28].	A reflector which uses micro-impulse infra-red radar [31].	Non-radioactive, wireless, and can be deployed at the time of biopsy. Does not interfere with MRI. Audiovisual reading.	High cost. Inability to adjust the position of the marker once deployed. Inability to generate audible signals at excessive depths. Larger than Magseed [31].

## Data Availability

The datasets generated in this study are publicly available in this open access publication without any restrictions.
